# Adoption of hand tractor technology in terrace farming: Evidence from the Hindu Kush Himalayan (HKH), Pakistan

**DOI:** 10.1016/j.heliyon.2023.e14150

**Published:** 2023-02-28

**Authors:** Ayat Ullah, Ashfaq Ahmad Shah, Miroslava Bavorova, Giri Prasad Kandel, Harald Kächele

**Affiliations:** a Faculty of Tropical AgriSciences, Czech University of Life Sciences Prague, Kamycka 129, 16500, Praha, Suchdol, Czech Republic; b Research Area 2 “Land Use and Governance”, Working Group: Sustainable Land Use in Developing Countries, Leibniz Centre for Agricultural Landscape Research (ZALF), Eberswalder Straße 84, 15374 Müncheberg, Germany; c Research Center for Environment and Society, Hohai University, Nanjing 210098, China; d Eberswalde University for Sustainable Development, Schicklerstraße 5, 16225 Eberswalde, Germany

**Keywords:** Hand tractor technology, Binary logit model, Extension contact, Communication networks

## Abstract

Adoption of improved agricultural technologies can help achieve the two Sustainable Development Goals (SDGs) of no poverty and zero hunger by 2030. This study investigates the determinants of farmers' adoption of hand tractors in the HKH region of Pakistan using binary logit model. We also examine what facilitates and what impedes the adoption of hand tractor adoption using key informant interviews. Results show that household head education, farming experience, knowledge of hand tractor use, access to credit, extension contact, and trust in technology positively affect the adoption of hand tractor; however farm size is negatively related. The findings reveal that ethnic conflicts, political conflicts, elite capture in decision making, unavailability of functional community-based entities, weak extension-farmers contact, as well as weak inter- and intra-community linkages are key barriers affecting hand tractor adoption. Similarly, observed changes on neighbors’ fields, experiencing hand tractor on trial basis, communication networks, risk observations, and trust propensities motivate hand tractor adoption in the study region. Thus, to effectively disseminate improved agricultural technologies, policymakers should consider these factors.

## 1 Introduction

The world faces challenges achieving the Sustainable Development Goals (SDGs) of zero hunger and ending poverty by 2030 [1,2]. Many inhabitants of developing countries are trapped in poverty, with most of their population being food insecure [2]. Since developing countries are heavily dependent on the agriculture sector for livelihoods [3], growth in agriculture can improve rural livelihoods and ensure economic growth, especially in developing countries [4]. The farmers’ adoption of modern agricultural technologies is important and cannot be ignored [5]. Adoption of agricultural technologies supports crop productivity, fosters employment creation, and improves household access to food [6]. Thus, the adoption of modern agricultural technologies (innovations) is an important factor affecting the welfare of farmers and playing a role in achieving zero hunger [7]. Development of agriculture sector through the adoption of modern technology is key for ensuring zero hunger and poverty alleviation [8].

Technologies have been developed and its dissemination has been ensured in farming communities for practical benefits on a regular basis [9]. However, the literature suggests that farmers in mountainous regions have not benefited from agricultural technologies, especially hand tractor technology [10,11]. The low benefits of hand tractor technology in the mountainous regions are due to, in part, the failure of initiatives aimed at promoting such technologies among terrace farming communities [12,13]. Thus, in mountainous regions, a large proportion of farming communities have not adopted improved technologies, thus affecting agriculture production, food security, and the welfare of the masses [14]. Hand tractors can cope with complex working conditions in mountainous regions and play an important role in terrace farm operations [15]. Adoption of conventional tillage operations, such as hand tractor technology, among small farm holders in terrace farming is important for food security and welfare [16]. Due to geographical and economic conditions, investment in new technology is difficult for mountainous farmers, which results in non-adoption of many important technologies [17].

It is particularly important to focus on how to promote technology adoption, especially hand tractors in hilly areas. To increase technology adoption among farmers, thus encouraging sustainable crop production, policy makers need to understand how to improve the conditions under which they disseminate technologies [18]. In this sense, understanding those factors that inherently facilitate or hinder agriculture technology adoption by farmers is important [19]. Researchers have identified various reasons justifying the limited adoption of technology by farming communities. For instance, some studies suggest farmers’ weak socio-economic conditions [11] and the prevalence of information asymmetry in input markets as important factors limiting technology adoption [20]. Other studies suggest that technological characteristics, like its innate complexity or compatibility, are important for its adoption or non-adoption alongside its welfare effects [21]. Studies also show that weak innovation systems reduce technology adoption whereas effective social networks inside a community increases its adoption and welfare effects [19]. Similarly, other initiatives, like extension programs, promote the adoption of various technologies through on-farm demonstrations in many regions [22].

Most existing studies focus on the adoption of improved seeds [5,23], precision agricultural technologies [20], climate change adaptation strategies [21], and sustainable vegetable production technologies [24] for improving crop production. To the best of our knowledge, no studies focus on the adoption and welfare effects of hand tractor technology diffusion across terrace farming in mountainous regions around the world. Hand tractor adoption across rigid mountainous regions, if promoted, is expected to enhance agricultural productivity, income, and welfare of farming communities. Thus, this study explores those factors that facilitate or hinder the conventional ways of tillage in traditional agriculture, such as adoption of hand tractor in terrace farming across mountainous regions of the world using the Hindu Kush Himalayan (HKH) region of Pakistan as its case study. Therefore, this study contributes to the identification of innovative factors and rational ways of agricultural technology adoption at household and community levels in mountainous regions. Identification of the determining factors underlying hand tractors will promote its adoption, thus resulting in increased crop productivity, enhanced food security, and improved rural livelihoods, while facilitating a systematic transition to sustainability. This study pursues the following specific objectives:

I. To identify those factors that affect adoption of conventional ways of tillage in traditional agriculture (such as hand tractor technology) in the HKH region of Pakistan; and
II. To compare the welfare effect of hand tractor adoption in the HKH region of Pakistan.

## 2 Materials and methods

### 2.1 Study area

**Fig. 1:**
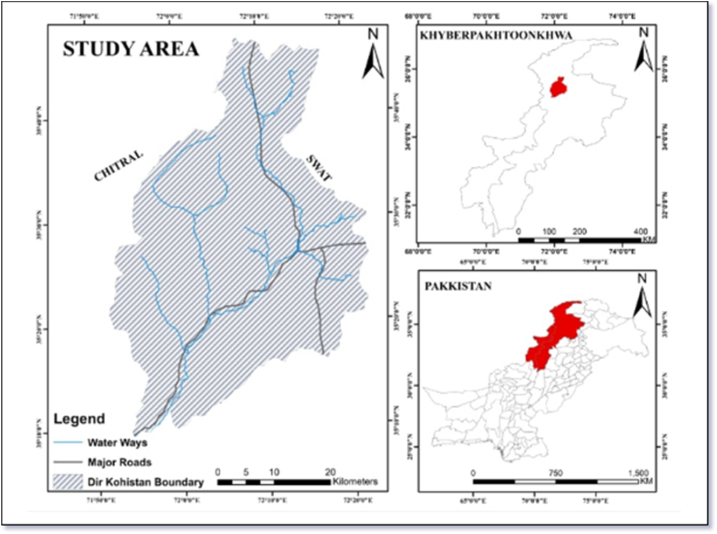
Map of the study area.

The Dir Kohistan region, located in the Hindu Kush Himalayan (HKH) Mountains, is a harsh hilly high land area consisting of agricultural landscape, forest resources, and residential areas (Fig. 1). The Dir Kohistan (350-9′ and 350-47′N latitude and 710-52′ to 720-22′E longitude) covers an area of 412,570 acres or 645 square miles (1670.54 square kilometers) and is in the north of the Dir Upper District of Khyber Pakhtunkhwa (KP) province. With a mean annual temperature ranging between 0 and 32 °C, the study area has an altitude ranging between 1677 and 5750 m. Due to its high altitude, the study area is characterized by frequent rainfall, with its average annual rainfall between 1000 and 1600 mm. With fertile croplands, farmers in the study area usually grow maize, wheat, beans, potatoes, and vegetables. Farming mainly comprises terrace cultivation in rain-fed conditions. Due to terrace farming, the main problems in the Dir Kohistan region include a decline in soil fertility, decreasing crop land, and limited use (due to low adoption) of agricultural technologies [10].

### 2.2 Data sources

#### 2.2.1 Households survey

To obtain background information on farmers’ socioeconomic, farm, and institutional dynamics, data were collected from 300 respondents in the Dir Kohistan region between February and April 2021. A list of farmers in the Dir-Kohistan valley was prepared with the support of the local agricultural extension department and households were selected from that list randomly. A comprehensive questionnaire was used for the data collection. The questionnaire was developed by experts and pretested by trained enumerators for necessary corrections in the field. In most cases, the data were collected from the heads of households by trained enumerators under the close supervision of the lead author from the study region. A total of 8 enumerators speaking the local languages were used for data collection. Enumerators speaking the local language were used because the study purpose was to explain to each respondent in their local language the purpose of the study and to obtain their written consent. Additionally, it was easy for local enumerators to collect data because each of them was known and trusted by the farming households in the region. Moreover, since 2018, the lead author is working a community development officer in the area, thus helping to smooth the collection of data for this study.

#### 2.2.2 Key informant interviews

In May and June 2021, we interviewed 60 key informants, including community leaders, community elders, community development workers, and agriculture/forest department officials following a quota sampling technique. A total of 15 experts were interviewed from each group using semi structured interviews. They had the focus on local farming communities. Prior to interviews, all key informants were contacted by the lead author. A proposed time and date were discussed with each key informant for the interview. During the invitation, key informants were presented with the background and purpose of the interviews. The lead author carried out all interviews as he was working as a community development officer in the study region and all the key informants knew and trusted him since he was in frequent contact with them throughout his stay in the region. The interview protocol comprised seven main questions exploring the key informants’ perceptions regarding the adoption/non-adoption of hand tractor/modern agricultural technologies in the study region:

1. What difficulties did farmers face in adopting hand tractor technology in the study region?
2. What motivates farmers' adoption of hand tractor technology in the study region?
3. What is the role of community and political conflicts in the dissemination of hand tractor technology?
4. What is the situation of intercommunity linkage and information sharing on modern agricultural technologies, including the hand tractor?
5. What is the perceived role of, or problems with, government institutions in the diffusion of innovations?
6. What is the role of elites in technology promotion in their communities?
7. What is the role of neighbors in technology promotion among non-adopters?

Interviews were recorded with a tape recorder and were transcribed in Urdu. From Urdu, each interview was translated into English by four independent interpreters.

### 2.3 Data analysis

The transcripts of the recorded key informants' interviews were analyzed using qualitative content analysis. Major topics and keywords from transcripts of interviews were tagged and subjected to word frequency count using a Microsoft Excel spreadsheet. We used frequency count to understand why mountainous farmers do not adopt the hand tractor and to reveal potential constraints that farmers face in adopting modern technologies. To ensure the key informants' anonymity, respondents from community leaders' group are represented as CL, respondents from community elders’ group are represented as CE, respondents from community development workers are represented as CDW, and the respondents from agriculture/forest department officials are represented as AO.

The quantitative data were analyzed using Statistical Package for Social Science (SPSS) version 25. The binary logistic regression model is considered as the appropriate model for analysis when dependent variable is dummy form [22,25]. Therefore, we used binary logistic regression to identify those factors affecting mountainous farmers’ adoption of hand tractor in the HKH region of Pakistan. Our dependent variable is a dichotomous phenomena that is equal to 1 if the farmer has adopted hand tractor technology and 0 otherwise. The binary logit model is widely used in similar studies across different disciplines (25, 26, 27).

The basic form of the logit model is shown in Eq. (1).

(1) $$Logit\left(pi\right)=In\;\left(\frac{pi}{1-pi}\right)=\beta_{0}+\beta_{1}x_{1,i}+\ldots +\beta_{j}x_{j,i}$$

where (pi) represents the probability of observation i (farmer) adoption of hand tractor and $x_{ji}$, is the value of the $j^{th}$ independent variable for the $i^{th}$ observation.

### 2.4 Selection of variables and expected outcomes

As the socioeconomic, farm, and institutional related variables of farmers are likely to influence hand tractor adoption, these are included in the study. The age of the farmer may influence agricultural technology adoption positively since elderly farmers are the key decision makers in the study region and all extension support is primarily delivered to old aged farmers in the study region [22]. Illiterate farmers are often more constrained in terms of access to, and understanding of, new machinery; thus, they usually do not adopt new technologies [22]. In contrast, adoption of new technologies among educated farmers is usually greater [23]. Previous studies reported negative correlation between family size and adaptation of new agricultural technologies [22,24]. Farmers with larger families typically appear to be rather traditional, unaware of the existence of new technologies, and usually not adopting such innovations [22]. Farming experience is also reported in many studies to be an important variable that positively affects adoption of improved agricultural technologies [28,29]. The variable of farm size included in the previous studies suggest that technologies are often adopted by farmers with larger farms, since farm size reflects households' asset ownership [19,30]. To use a hand tractor, a farmer should have relevant knowledge and skills. Many previous studies stress the building of farmers' knowledge on a particular technology for its adoption [31,32]. Access to information is also an important variable that positively affects agricultural technology adoption [19,33]. Empirical studies show a mixed effect of farmers' participation in off-farm employment and their adoption of agricultural technologies [34,35]. Gedikoglu et al. [34] show a positive influence of off-farm employment, whereas Suvedi et al. [35] show a negative effect of off-farm employment on agricultural technology adoption. Therefore, in this study, the off-farm employment could either positively or negatively influence hand tractor adoption in the study region. Access to credit for agriculture and extension contact both indicate farmers' institutional network. Empirical studies show that access to credit and extension contact positively influence farmers' adoption of new agricultural technologies [33,36]. Both variables are included because research associates these with the adoption of improved agricultural technologies. Elite capture in decision making is also an important variable that negatively affects farmers' adoption of new agricultural practices [37]. Farmers’ subjective trust in hand tractor technology is divided into two groups. The first group comprises those farmers who believe that technology plays an integral role in enhancing agricultural productivity and income, while the second group includes those farmers who believe that agricultural technology adoption will not contribute to increased agricultural productivity. The role of trust on improved agricultural technologies in increasing agricultural productivity and income is widely explored [38,39]. Trust that agricultural technology will fulfill household needs is likely to increase the likelihood that a farmer will adopt hand tractors. Farmers who trust that improved agricultural technologies will increase agricultural productivity and fulfill household needs are more likely to adopt hand tractor technology than those who do not. Therefore, trust of hand tractor technology is included in the study, to be positively correlated with hand tractor technology adoption.

## 3 Results and discussion

### 3.1 Descriptive statistics

Table 1 shows definitions and summary statistics of the variables used in the logit analysis. About 35% of the respondents adopted hand tractor technology in the study region (Table 1). Our analysis shows no significant differences in the ages of adopting and non-adopting farmers. The study shows significant differences in education among adopting and non-adopting farmers. Our descriptive statistics do not show any significant differences between the family sizes of hand tractor technology adopters and non-adopters. There are significant differences based on farm sizes between the adopters and non-adopters of hand tractor technology in the study region (Table 1). Our descriptive statistics show that 45% of adopters and 63% of non-adopters had knowledge of hand tractor use (Table 1). There are significant differences between the adopters and non-adopters of hand tractor technology concerning the knowledge of hand tractor use. Our study shows that 31% of adopters and 29% non-adopters had access to information (Table 1). In our study, 56% of adopters and 60% of non-adopters show participation in off-farm employment (Table 1). In this study, 49% of adopters show access to credit and 18% have extension contacts. However, extension contact is significantly different between adopters and non-adopters (Table 1). In our study, 50% of adopters and 53% of non-adopters show elite capture in decision making in their communities (Table 1). Results show that 74% of the adopters trust that improved agricultural technologies will increase agricultural productivity and fulfill household needs (Table 1).

**Table 1:** Description of the variables used in the binary logit model.

Variables	Type of measurement	Expected signs	Adopters (S.D)	Non-adopters (S.D)	t-value
y – Adoption of hand tractor	Dummy (1 if yes, 0 if no)				
$X_{1}$– Age	Numeric (Years)	+	47.74 (12.88)	46.69 (11.23)	.73
$X_{2}$– Education	Numeric (Years)	+	2.64 (4.41)	4.77 (5.72)	−3.32***
$X_{3}$– Family size	Numeric (number)	−	12.30 (5.57)	12.54 (6.50)	−.31
$X_{4}$– Farming Experience	Numeric (Years)	+	27.70 (12.19)	29.30 (12.97)	−1.04
$X_{5}$– Farm size	Numeric (Acre)	+	4.98 (3.93)	2.19 (2.31)	7.70***
$X_{6}$– Knowledge of hand tractor use	Dummy (1 if yes, 0 if no)	+	0.45 (0.50)	0.63 (0.48)	−2.92***
$X_{7}$– Access to information	Dummy (1 if yes, 0 if no)	+	0.31 (0.46)	0.29 (0.45)	.30
$X_{8}$– Participation in off-farm employment	Dummy (1 if yes, 0 if no)	±	0.56 (0.49)	0.60 (0.49)	−.72
$X_{9}$– Access to credit	Dummy (1 if yes, 0 if no)	+	0.49 (0.50)	.44 (0.49)	.81
$X_{10}$– Elite capture in decision making	Dummy (1 if yes, 0 if no)	−	0.50 (0.50)	0.53 (0.50)	−.47
$X_{11}$– Extension contact	Dummy (1 if yes, 0 if no)	+	0.18 (0.38)	0.34 (0.47)	−3.00***
$X_{12}$– Trust on technology	Dummy (1 if yes, 0 if no)	+	0.74 (0.43)	0.03 (0.19)	−15.70***

*** denotes statistical significance at the 1% level.

Adopters comprise 105 (35%) and non-adopters 195 (65%) of the total sample.

### 3.2 Respondents’ attitudes towards farming (profitability comparison among adopters and non-adopters of hand tractor)

**Fig. 2:**
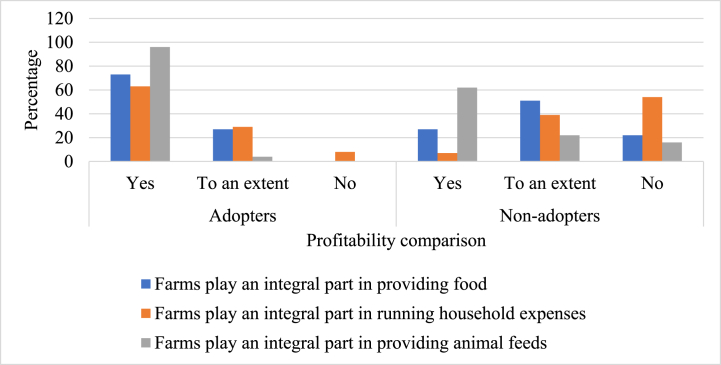
Profitability comparison among adopters and non-adopters. Note: Our questionnaire contained questions on profitability that facilitates this comparison between adopters and non-adopters, including their per acre yield, per acre inputs cost, per acre laborer/hand tractor costs, and net profit. However, more than half of the respondents were not able or were not willing to answer these questions. Therefore, we modified our questions into the current form.

Regarding the general attitude toward farming among the adopters and non-adopters of hand tractor technology, our results reveal that the majority (73%) of the adopting respondents believe that farming plays an integral part in providing food to their families (Fig. 2). In contrast, most of the non-adopting respondents (73%) report that their farming does not play an integral role in providing sufficient food for their families (Fig. 2). Most adopting farmers (63%) report that, due to increased production and improvement in their farming because of modern technology adoption, their farms are covering their household expenses (Fig. 2). In contrast, most non-adopters (93%) report that their farming is not sufficient for household expenses and many household members are working as wage labor to help meet household expenses (Fig. 2). Regarding the farm's role for household animal welfare, adopters (96%) are more likely than non-adopters (62%) to report that the farm plays a potential role in providing animal feed throughout the year (Fig. 2). Adopters agree that the farm's role in their lives signifies the importance of modern technology in farming. All the adopting farmers report that the adoption of modern technologies, including hand tractor technology, has brought improvement to their farming, which resulted in improvements in the quality of life of their lives. These results agree with the previous findings of Pfeiffer et al. [40], who reports that adoption of modern agricultural technologies increases household welfare, which further improves the attitudes of adopters toward the benefits of technology adoption. In other parts of the world, technology adoption by smallholder farmers has played an important role in providing food, income, and employment in the crop and livestock sector under similar conditions [41,42]. This means that policymakers can increase income of households by increasing adoption of modern agricultural technologies.

### 3.3 Logit model results

Identification of the socio-economic, farm, and institutional characteristics of hand tractor adoption not only helps to better understand the relationship between the socio-economic factors and improved agricultural technology adoption but also helps to formulate strategies that address those issues inhibiting the adoption of modern technologies in the HKH region of Pakistan. As described in the methodology section, a total of 12 variables are analyzed using a binary logistic regression model to examine their relationship with hand tractor adoption at the household level. The −2 log-likelihood value for data in the model is 179.26 and the R2 value is 0.692, indicating fitness of the model. We validate the performance of our binary logit models using the receiver operating characteristics (ROC) technique, which is a commonly employed method for assessing the performance of binary classifiers [10]. The area under the ROC curve for the adoption of hand tractor technology in the HKH region is 0.84, which falls in the excellent model performance range. The Hosmer and Lemeshow test value for the binary logistic regression is 0.301, indicating no significant difference between the predicted value and the observed value, specifying a good fit of the model. To test multicollinearity in the model, variance inflation factors (VIFs) were calculated, with all resulting values being smaller than 10 (ranging from 1.065 to 2.964). A VIFs value smaller than 10 suggests that there is no multicollinearity in the model [10]. The statistical significance of the parameters of hand tractor adoption included in the logistic regression model is shown in Table 2. Of the 12 variables hypothesized to affect hand tractor adoption in the mountainous regions of KP, seven are found to be significant.

**Table 2:** Results of the Logit model.

Variables	Coefficient	Standard error	Wald χ2	Sig	Odds ratio
Age	.005	.020	.069	.793	1.005
Education	.082	.043	3.602	.058	1.086
Family size	.021	.035	.350	.554	1.021
Farming Experience	.035	.016	4.802	.028	1.035
Farm size	−.298	.073	16.478	.000	.742
Knowledge of hand tractor use	1.670	.897	3.466	.053	5.312
Access to information	.020	.458	.002	.965	1.020
Participation in off-farm employment	.310	.423	.535	.464	1.363
Access to credit	.754	.432	3.044	.081	2.126
Elite capture in decision making	−.652	.893	.534	.465	.521
Extension contact	1.023	.582	3.087	.079	2.781
Trust on technology	4.524	.605	55.910	.000	92.165

Summary statistics: −2 Log-Likelihood = 179.26; Pseudo-R2 = 0.692; Prob > χ2: 0.00, Hosmer and Lemeshow test = 0.301.

The variable household head education is positively associated with the adoption of hand tractor technology. A one unit increase in households' head education increases their probability of hand tractor technology adoption by 0.86%. The probable reason could be the fact that educated farmers have better access to, and understanding of, information about different agricultural technologies. They understand the value of adopting modern technologies better than illiterate farmers. Studies by Rakgase and Norris [43] and Islam et al. [44] in other parts of the world find that education has a significant positive influence on the adoption of different types of agricultural technologies and practices, similar to the results of this study. Likewise, the variable of farming experience also has a significant positive relationship with farmer's adoption of hand tractor technology. A one unit increase in farming experience increases the household's probability of hand tractor technology adoption by 0.35%. This means that the greater the experience of a farmer in agriculture, the more likely he/she is to adopt hand tractor technology. The probable reason could be that as farmers accumulate experience, they switched from traditional to modern farming technologies on the basis of observed performance of modern technology and by learning by doing. This means that policymakers could enhance dissemination of modern technologies by focusing on experienced farmers in a community. Previous studies [45,46] from other parts of the world report similar findings, as they find that as farming experience increases, the probability that a famer adopts technology increases in North America, South America, and China. The variable “farm size” has a significant and negative relationship with the adoption of hand tractor technology by mountainous famers that is beyond our expectations. Our results demonstrate that with one unit increase in farm size of a farmer, the 7.42% less likely he will adopt a hand tractor in agriculture. In contrast to our findings, results in other parts of the world suggest that the farm size variable is significantly and positively related to technology adoption, with farmers who have large land holdings being more likely to adopt improved agricultural technologies [26,33]. The negative association of farm size with hand tractor adoption in our study might be due to low acreage (land holding size) possession by farmers in the study region. The area is rather mountainous and farmers do not have large land holding sizes in the study region [27]. Much of the research suggests that small farm sizes, along with cultivation on steep slopes, reduces the probability of adopting improved agricultural technologies due to the low possibilities for mechanization in such regions [[47], [48], [49]]. The other variable with a significant positive effect on hand tractor adoption is knowledge about the use or management of hand tractors. A one unit increase in a farmer's knowledge of hand tractor use increases the probability of his/her hand tractor adoption by 3.12%. This means that those farmers who have knowledge about hand tractor use and management have adopted it. The policy implications could be that extension agents should update farmers' knowledge on the new technology usage and management for enhanced adoption. In the study area, farmers have low educational levels, with limited access to skills enhancing programs, and they are not aware of mechanized technologies [22]. Lack of knowledge about the use of modern mechanization hinders its adoption and proper use. Like our study, research from other parts of the world suggests that knowledge about the use of mechanized technology and its management positively affects its adoption [50,51]. The other variable with a significant positive effect on hand tractor adoption is access to agricultural credit. A one unit increase in households' access to credit increases their probability of hand tractor technology adoption by 1.26%. In the study region, farming households with access to agricultural credit are more inclined to adopt modern technology. Lack of access to agricultural credit hinders adoption of hand tractor technology and can reduce crop productivity. Similarly, existing research reports a positive effect of access to agricultural credit on the adoption of improved agricultural technologies, including hand tractor technology [33,52]. Access to credit induces farmers to invest in improved agricultural technologies and, hence, plays an important role in its adoption [33]. Studies suggest easy access to credit is a policy that enhances farmers' adoption of modern technologies [06, 52]. An important variable affecting hand tractor adoption significantly and positively is extension contact. A one unit increase in households' contact with extension agent increases their probability of hand tractor technology adoption by 7.81%. This means that the farmers who had contact with extension agents adopted hand tractor technology in the study region. The findings by Yigezu et al. [53] and Abegunde et al. [54] from Sub Saharan Africa agree with our results: they report that extension contact positively affects adoption of agricultural technologies by farmers. The policy could be more frequent extension agent contact with farming communities and households in order to convince them to adopt agricultural technologies. The other variable with a positive and significant effect on agricultural technology adoption is trust in technology. Technology trust, including farmers' belief in the relevancy of a technology to a farmer's needs, reliability and its importance in farm households' food security will more likely boost its adoption. Therefore, the policy implication is to build farmers' trust in hand tractor technology to facilitate its adoption. The findings of Chalaganidze et al. [55], Lin et al. [38], and Baiyegunhi et al. [39] agree with our results as they report that farmer's trust in agricultural technology positively affects its adoption.

### 3.4 Results of key informants’ interviews

#### 3.4.1 Factors hindering community members’ adoption

##### 3.4.1.1 Ethnic conflicts in society

**Fig. 3:**
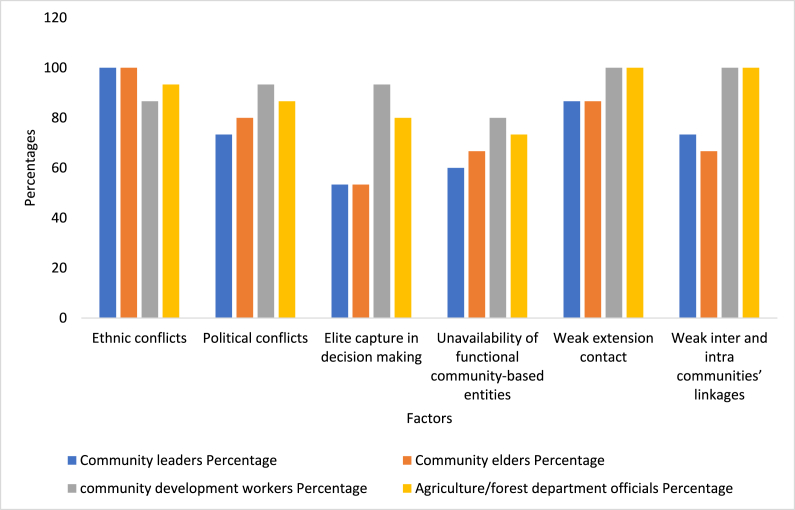
Barriers to hand tractor adoption.

Presented with a list of key obstacles hindering the adoption of hand tractor technology, 100% of community leaders, 100% community elders, 86.66% of community development workers, and 93.33% of agriculture/forest department officials chose ethnic conflicts inside the communities as a major barrier to hand tractor adoption (Fig. 3). According to our results, ethnic conflicts inside the community are a crucial obstacle in the diffusion of hand tractor technology for the agricultural extension department, resulting in meager adoption of hand tractors by farmers. Key informant interviews reveal that, in the study region, farmers residing in disputed situations in their villages mostly due to differences in their ethnicities. Nearly in every village in the HKH in Pakistan, the 3 primary ethnic groups including Pashtun, Gujjer and Kohistani reside, and they have competing claims on crop lands and the ownership of other resources that are required for farming [56]. These disputes result in a resistance to change as well as in the non-adoption of improved technology and practices, thus exposing the area to increased crop land degradation, as previous studies report [10,56]. Since the disputed communities and farmers reside next to each other, their respective crop lands are near the other disputed communities and farmers houses (and away from their own communities). Therefore, if a farmer from one community enters their respective crop lands or adopts modern technology for restoring degraded lands, he must pass through the household settlements of their opponent communities along with the technology (which reveals the lands restoring intentions of a farmer to his opponent community), which is a challenging task. Many key informants report that:

*Adoption of hand tractor technology is, in principle, readily available – Technology that can restore degraded hilly lands. But the restoration of lands and adoption of hand tractor technology is a difficult step in the face of associated ethnic differences and conflicts in the area* CL 13, 15, CE 7, 11, CDW, 3, 5, 6, 10, AO 4, 5, 11, 15

Similarly, some key informants report that:

*The benefits of adopting hand tractor technology is evident in the hilly regions; however, the mixed ethnicities that reside in the villages do not allow for its adoption.* CL 1, 2, CE 4, 15, CDW 1, 2, 8, 9, AO 2, 6, 14

Such insights are also supported by Ullah et al. [10] and Biland et al. [56], who find that ethnic conflicts severely impact communities’ adoption of novel practices and technologies, thus negatively affecting communities landscape restoration practices.

##### 3.4.1.2 Political conflicts

Key informants note that political differences within communities also create divisions among farmers that negatively affect household adoption of hand tractors, crop lands restoration, and, ultimately, economic benefits from the agriculture sector (Fig. 3). Most key informants (73.33% of community leaders, 80.00% community elders, 93.33% of community development workers, and 86.66% of agriculture/forest department officials) specify that political conflicts inside the community are a crucial obstacle, indicating that extension services could facilitate adoption in the short run by delivering extension services free from political influences. A key informant reports that:

*For a long time, political divisions and conflicts have been influencing extension services and the diffusion of innovations to farming communities. Political groups influence farmers' decision making. They divert all benefits from extension and community-based organizations to their own voters. Political groups are creating problems in delivering services to the farmers that do not follow their ideologies or groups. These political groups influence farmers' activities and create problems in the smooth run of different extension tasks*. CE 2, 3, 8, CDW, 1, 5, 11, AO 6, 13, 15

Like this study, Rotz et al. [9] and Newell [57], studying Latin America, report that political divisions create conflicts over technology adoption and agricultural futures in communities. They report that political interference affects technology adoption, creating hurdles for agricultural development by adversely affecting the adoption and the livelihood activities of farming communities.

##### 3.4.1.3 Elite capture in decision making

Results from our key informant interviews reveal that 53.33% of community leaders, 53.33% community elders, 93.33% of community development workers, and 80.00% of agriculture/forest department officials report that the adoption of agricultural technology, like hand tractors, is also heavily influenced by elite capture within communities (Fig. 3). According to our results, the elites have completely captured the decision-making positions, both at the community and household levels, having become the only ones to make decisions on whether or not to adopt any given technology. Elite capture is an important obstacle hindering the diffusion and adoption of hand tractors in the study region. Many key informants report that:

*Since the start of different landscape restoration projects, the rich people in our regions have become more powerful and involved in village developmental activities. The powerful elite influence the adoption of agricultural technologies in their villages. We have repeatedly noticed and have found strong evidence of elite capture in almost every land restoration activity, including the adoption of hand tractor technology – especially in traditional, remote, and illiterate communities. The elite mostly make inappropriate decisions and they do not allow poor communities to restore their lands, rather making the poor work only on their lands on a tenancy basis.* CL 5, 7, CE 2, 4, 9, 11, 13

The elite gives preference to their own lands and resources, wanting the resource poor farmers to only work on their lands, not allowing them to make decisions on technology adoption or their own lands cultivation. The policy implication is that extension agents should establish good relationships with ordinary farmers and help them technically. Direct information to farmers and encouraging farmers to understand and adopt technology will reduce elite capture in decision making. The literature suggests that elite capture is common around the world, restricting the adoption of innovations by small farmers in many communities around the world. For instance, Pan and Christiaensen [58], and Ullah et al. [37], report that decision making regarding technology and improved agricultural practices adoption is inherently captured by the elites of communities and that the most benefits of landscape restoration initiatives are unequally distributed due to elite capture.

##### 3.4.1.4 Unavailability of functional community-based entities

The unavailability of functional community-based entities, like village development committees, is also ranked as a top obstacle inhibiting the adoption of improved agricultural technologies (Fig. 3). About 60.00% of community leaders, 66.66% community elders, 80.00% of community development workers, and 73.33% of agriculture/forest department officials claim that the lack of functional community-based entities is an important variable negatively affecting hand tractor adoption in the HKH region of Pakistan. Functional community-based entities act as a focal point for the general community in adopting sustainable agricultural technologies [59]. Many key informants reveal that:

*The adoption of hand tractor technology is limited due to non-availability of functional community-based entities in the region. Establishment of community-based organizations is important for technology dissemination in such isolated regions.* CE 3, 7, CL 11, 13, CWO 1, 6, 7, 14, AO 2, 7, 9, 12

This indicates that policy should facilitate the establishment of community-based organizations at the village level across the region. Establishing community-based organizations can provide a platform for better communication of research results and productivity enhancing technologies to farmers, thus increasing adoption of modern technology. Community based organizations can make farmers aware of the possible technology and their role in meeting farmer's needs. Many key informants (elders and local leaders) report:

*Hand tractor technology is necessary for farming in hilly areas and the effect of community-based organization is important for adoption.* CE 4, 5, 6

Existing studies confirm what is found in our study: the literature concerning innovation diffusion suggests that community-based organizations are important for technology adoption by farm households in many countries [52,60].

##### 3.4.1.5 Weak extension contacts

The key informant interviews also show that weak extension-farmer contact is also a potential barrier for the adoption of improved agricultural technologies, including hand tractors, by mountainous farmers (Fig. 3). About 86.66% of community leaders, 86.66% community elders, 100% of community development workers, and 100% of agriculture/forest department officials claim that weak extension-farmer contact is responsible for farmers’ non-adoption of hand tractor technology in the study region. The key informants reveal that:

*Frequent extension-farmer contacts are vital for persuading farmers of the merits of the new technology (hand tractor) and ensuring its large-scale adoption. Unfortunately, this important link is missing completely in our region. We do not have extension agents and the extension department office is poorly equipped. The public extension department hardly plays any role in dissemination awareness and the diffusion of hand tractor technology*. CL 1,3, 4, CE, 6, 7, 9, 14, CDW 1, 3, AO 2, 5, 9

This is in line with Faborode and Ajayi [61], and Oyinbo et al. [62], who note that weak extension delivery has resulted in poor awareness and adoption of modern technology by farmers in Africa. This suggests that policy makers can enhance farmers’ adoption of hand tractor technology by improving links between farmers and agricultural extension agents in HKH.

##### 3.4.1.6 Weak inter- and intra-community networks

Fig. 3 shows the perceptions of key informants regarding weak intercommunity contacts and networks, along with how it inhibits the adoption of hand tractor technology across the mountainous regions of Pakistan. About 73.33% of community leaders, 66.66% community elders, 100% of community development workers, and 100% of agriculture/forest department officials report that weak inter- and intra-community networks are responsible for farmers non-adoption of hand tractor technology in the study region. The key informants report that:

*In mountainous regions, communities reside in isolated conditions. The villages are on peaks. It takes an entire day to travel from one village (that often resides on one peak) to another village (that most often resides on another peak). I is not only inter community networks that are poor, but extension contact – all because of the situations of villages and population in mountainous regions. In order to reach all communities, the extension department needs hundreds of agents to provide extension services on improved agricultural technologies. The poor inter community networks results in both low awareness and limited information among farming households and communities with respect to the existence of improved agricultural technologies*. CL 1, 3, 14, CE, 6, 7, 9, 14, CDW 1, 3, AO 2, 5, 9

The key informants reveal that the poor inter-community network is further affected by frequent conflicts between communities. Many key informants report that:

*Conflicts exist among communities due to common differences among groups which weaken contacts among them. Thus, farmers do not share information on improving agricultural technology and farming.* CL 2, 7, 12, CE, 6, 11, 12, 13, CDW 2, 3, AO 4, 6, 10

Since the inter- and intra-community networks are a main factor undermining hand tractor technology adoption, the policy implication is that the extension system should establish an inter-community network. Findings by Ullah et al. [5] and Ullah et al. [19] in this region also report poor inter community contacts and networks in the region, as well as its reciprocal effects on low or non-adoption of agricultural technology.

#### 3.4.2 Factors motivating community members’ adoption

##### 3.4.2.1 Observed changes on neighbors’ fields

**Fig. 4:**
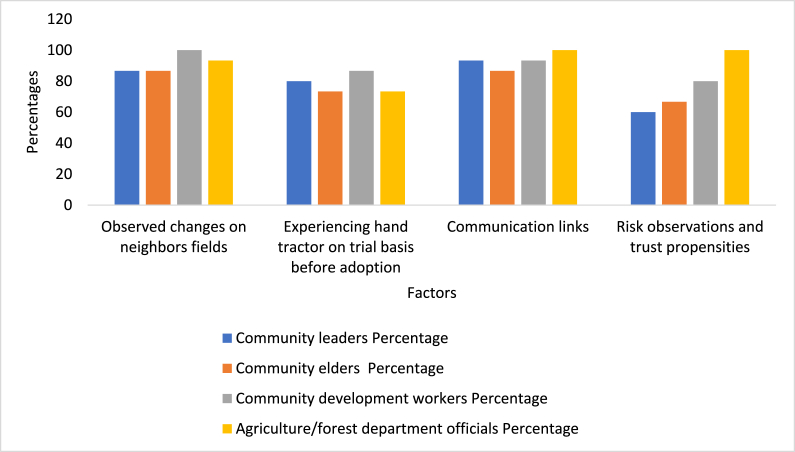
Motivating factors of hand tractor adoption.

Most of the participants (86.66% of community leaders, 86.66% community elders, 100% of community development workers, and 93.33% of agriculture/forest department officials) report that, in the study area, many non-adopters farmers have noticed that the farmlands of adopting farmers are exhibiting progressive changes, with large sloped land areas being restored rapidly and inexpensively. Similarly, they clearly observe that hand tractor adoption supports people's livelihoods because it increases agricultural performance. The changes to the landscapes of adopting household allowed non-adopting households to understand the benefits of modern technologies like hand tractors. Fig. 4 presents a summary of the key motivators that turn non-adopters into adopters. Most key informants suggest that mobilizing neighboring farmers is necessary if sustainable hand tractor adoption among non-adopting households is to be achieved. Many key informants report that:

*In the past 5 to 10 years, I have witnessed changes in decisions of some non-adopters of hand tractors. I know that the decision of non-adopters to change were due to observed changes on adopters' fields. The non-adopters especially observed the areas restored by adopters, the yields of adopters, and the environmental benefits of hand tractors.* CL 1, 2, 3, 9, CE, 7, 8, 9, 14, CDW 13, AO 2, 5, 9

Similarly, some key informants report that

*Many years ago, the government extension department used to conduct field days where they promoted new technology. The field days were aimed at showing the benefits of technology adoption on fields. We observed that these field days were very effective in disseminating agricultural technologies to places where the farmers were completely unwilling to adopt such technologies. Now the government does not conduct agricultural field days and farmers do not attend them. The farmers adopting hand tractors see its benefits on adopters' farms and … the government needs to arrange frequent visits of farmers to areas where hand tractors have been successful. Regular field days and farmer visits to areas where the hand tractor has successfully been used can give farmers some new ideas and motivation to adopt hand tractors*. CL 4, 5, 6, 7, CE, 4, 5, 10, 12, CDW 1, 7, AO 14

Thus, a main factor shaping the motivation of farmers to adopt hand tractor technology is farmers observations of changes on adopters' fields. A policy that would facilitate and encourage the adoption of hand tractor technology in the HKH region of Pakistan and in other countries is to conduct demonstrations and provide field visits to regions where this improved technology is adopted and successful. Our results are similar to those of Meijer et al. [11], and Campbell and King [63], who find that perceived relative advantage of a new technology is related to its adoption in sub-Saharan Africa and the United States, respectively. Mariano et al. [64] conclude that efforts to raise farmers' observations of the effectiveness or success of innovative agricultural technologies on farmers’ fields is likely increase its adoption.

##### 3.4.2.2 Experiencing hand tractor on trial basis before adoption

Results from key informant interviews highlight that the government could enhance hand tractor diffusion by promoting on-farm trials before actual adoption. Many key informants (80.00% of community leaders, 73.33% community elders, 86.66% of community development workers, and 73.33% of agriculture/forest department officials) note that they have observed many reluctant farmers adopting hand tractors following a temporary field trial. This is also an important element of individual decisions about agricultural innovations or modern agricultural technologies. The intended benefits of limited first-time use, on a trial basis, and the trial stage often leads to adoption of innovations. Many key informants report:

*We have noted that the adoption of hand tractor technology, along with other innovations, like agroforestry and improved hybrid seeds, have increased dramatically after initial trials. However, most of the time, trails of innovations, especially of hand tractors by farmers, have been promoted by external agents, mostly extension workers. These days, government extension departments are encouraging direct adoption, which mostly fails. The public government extension department has completely abandoned, or drastically limited, field trails in mountainous regions these days.* CL 1, 2, 3, 9, CE, 7, 8, 9, 14

Literature on the adoption of innovations from many regions of the world suggests that the trail of a new technology on limited basis decreases farmers' uncertainty, thus enhancing its later adoption [65,66]. Even the government can motivate the adopting farmers to help non-adopters in trials, enhancing their adoption potentials [66]. The key message from previous studies is that the chances of trialing and adoption are highest if the new technology is perceived by farmers as highly relevant to their farm conditions and potential needs [64,67]. Information dissemination is necessary for developing farmers’ perceptions on the need for technology adoption in connection to their farm problems and prospects [5,68].

##### 3.4.2.3 Communication links

Our key informants (93.33% of community leaders, 86.66% community elders, 93.33% of community development workers, and 100% of agriculture/forest department officials) report that, in the study region, those farmers who communicate with, and are social influenced by, progressive farmers have adopted hand tractors. However, farmer connections are very rare, typically limited to very few neighboring farmers. Even here they do not frequently exchange ideas and information on technology and agriculture. Furthermore, non-adopters also use a very few sources of information in the study region, which affect their adoption of hand tractor technology. Many key informants report that adopting farmers have access to many more sources of information than do non-adopting farmers. Further adopting farmers are well connected to neighboring farmers and to farmers in other communities. Many key informants suggest that:

*Due to the rigid situation of the mountainous regions, social connectedness among farmers in the area is weak; it does not support the exchange of ideas on technologies. If the extension service is interested in dissemination of hand tractor technology, then they need to establish connections not just between farmers but also between farming communities. Through social connectedness, non-adopters will observe their neighbors' success from using hand tractors and they will also adopt hand tractor technology.* CDW 13, AO 2, 5, 9

Previous studies also report that effective communication links establish social influences among farmers, thus resulting in non-adopting farmers deciding to adopt successful practices [69,70]. Riley et al. [71] report that effective communication links among farming households facilitate the exchange of ideas and increase the adoption of modern technologies. Thus, a policy to enhance the adoption of hand tractor technology is to establish inter-connections among community members. Establishing inter-connections among community members will increase technology adoption in mountainous regions globally because it will help to overcome the physical distance innate to these areas.

##### 3.4.2.4 Risk observations and trust propensities

Our key informant interviews reveal that 60.00% of community leaders, 66.66% community elders, 80.00% of community development workers, and 100% of agriculture/forest department officials perceive that the farmers’ risk perception affects their hand tractor adoption, in particular with respect to cropland degradation, productivity losses, and their trust that hand tractors can help restore agricultural landscapes. They reveal that farmers are aware of the changes in croplands due to non-cultivation and delayed cultivation on sloped lands. The changes in agricultural lands that they observe in the sloped areas of mountainous regions are increasing farmers' adoption of modern technologies and practices, including hand tractors. The adopters realize that failing to adopt hand tractors will result in soil depletion and will negatively affect both agriculture and livelihoods. Many community leaders identify that:

*We have observed that the farmers in our communities have experienced a decline in soil fertility and an increase in soil erosion due to non-cultivation of large areas on sloped lands. The uneven rainfall, observed by farmers, always results in floods on uncultivated lands and in damage to houses and livestock damage. In some years, when they used hand tractors and cultivated sloped lands with crops and agroforestry, the damage was low and community livelihood better. In other years when they did not use hand tractors, they failed to cultivate lands and, when rainfall was higher than normal, soil erosion was common and the damages to their infrastructure was higher. Such observations have developed their trust in hand tractor technology and they know that they can only cultivate rigid sloped lands if they adopt hand tractors that increase productivity and livelihoods. Thus, they adopt hand tractors.* CDW 1, 5, 13, AO 12, 15

Our overall finding is that most key informants responding to our study acknowledged not just the risks related to agricultural productivity in sloped areas but also their trust that adopting hand tractors would help cope with these risks. Thus, hand tractor adoption has increased, consequently improving agricultural productivity and the livelihoods of farming households. This is similar to studies by Takahashi et al. [42] and Ullah et al. [72], who find that those farmers most affected by agricultural risks show evidence of more effective adoption initiatives than those farmers unaffected by risks associated with agriculture.

## 4 Conclusion and policy implications

The adoption of hand tractor technology in the HKH mountainous region can restore terrace farming in hilly areas, thus increasing crop yields, food security, and the income of farming households in Pakistan. Therefore, this study examines the subjective comparison of profitability among adopters and non-adopters of hand tractor technology, estimating those factors that affect the adoption of hand tractors for cultivation in terrace farming in hilly areas. We use household-level survey data collected from farmers alongside key informant interviews to identify those factors that influence the adoption of hand tractor technology in the mountainous region of KP and to recommend strategies that enhance adoption among farmers in Pakistan. Our results suggest that, among adopting farmers, farm outputs play an integral part in providing food, animal feed, and covering household expenses. Results of the binary logit model show that several factors explain the adoption and/or non-adoption of hand tractor technology by farmers in the HKH region of Pakistan. Positively related factors include household head education, households head farming experience, knowledge of hand tractor use, access to credit, extension contacts, and farmers trust in technology. In contrast, farm size negatively affects the adoption of hand tractor. As highlighted by our key informants' interviews in the HKH region, the main barriers to hand tractor adoption include ethnic conflicts, political conflicts, elite capture in decision making, unavailability of functional community-based entities, weak extension-farmers contact, as well as weak inter- and intra-community linkages. Similarly, observed changes in neighbors’ fields, experiencing hand tractors on trial basis before adoption, communication networks within and across communities, risk observations, and trust propensities motivate hand tractor adoption in the study region.

Policymakers must understand those factors that hinder the adoption of hand tractors in the study region. Here, high exposure to extension services and increased frequency of extension-farmer contacts are important for the promotion of hand tractor adoption in the study region.

This study also suggests that increasing the adoption of hand tractor technology could be facilitated by disseminating knowledge of hand tractor use while building confidence and trust in technology. As limited access to resources often underlie the non-adoption of hand tractor technology, access to credit for resource poor farmers is necessary. Developing inter- and intra-community links can also disseminate awareness of hand tractor technology, thus promoting its adoption. Further, improving extension services can reduce political and elite capture. Effective dissemination of hand tractor technology needs functional community-based entities to be established in mountainous communities. Finally, adoption of hand tractor technology among farming communities could be encouraged through group farming, with the help of educated and experienced farmers.

## Funding

The first author acknowledges the post-doctoral funding from the Czech University of Life Sciences Prague, Prague, Czech Republic, under the project OP VVV no. CZ.02.2.69/0.0/0.0/18_053/0016979 and the APC funding by the Open Access Fund of the Leibniz Association.
