# Supplementary material for: Adoption of hand tractor technology in terrace farming: Evidence from the Hindu Kush Himalayan (HKH), Pakistan
Source: Heliyon. 2023 Feb 28;9(3):e14150. doi: 10.1016/j.heliyon.2023.e14150 (PMC10018466; doi:10.1016/j.heliyon.2023.e14150)
Supplement: Multimedia component 1 [file mmc1.pdf]

Questionnaire No. [ ]

Household survey questionnaire for collecting data from farm households on their demographic, farm and institutional variables, perceptions, and factors driving their adoption of hand tractor technology

-	Are you the head of the household? Yes No (Continue only if yes)
	**Socio-economic/Personal data**
1	What is your name?
2	Contact phone numbers:
3	Name of village
4	How old are you?
5	How many people are living in your household?
6	What is your educational background?
7	Do you participate in off-farm employment? a. Yes b. No
	**Farm data**
8	Since when you have been involved in cultivation?
9	What is the total size of your farm?
10	Do you believe that farm play an integral part in providing food? a. Yes b. To an extent c. No
11	Do you believe that farm play an integral part in running household expenses? a. Yes b. To an extent c. No
12	Do you believe that farm play an integral part in providing animal feed? a. Yes b. To an extent c. No
13	What is your yield per acre?
14	What is your input cost per acre?
15	What is your labor/hand tractor cost per acre?
16	What is your net profit from the farm?
	**Machinery data**
17	Do you have access to information on farming and farm machinery? a. Yes b. No
18	Do you have relevant knowledge of using hand tractor technology? a. Yes b. No
19	Do you trust on farm machinery to meet your household needs? a. Yes b. No
	**Institutional data**

20	Do you have contact with extension agent?	a. Yes b. No
21	Do you have access to credit?	a. Yes b. No
	**Community level data**	
22	Do a household's head adoption have been affected by elite dominance in decision making?	a. Yes b. No
	**Dependent variable**	
23	What is your adoption status?	a. Adopter b. Non-adopter
